# Clinical Benefit of Long-Term Disease Control with Pomalidomide and Dexamethasone in Relapsed/Refractory Multiple Myeloma Patients

**DOI:** 10.3390/jcm8101695

**Published:** 2019-10-16

**Authors:** Marina Silvia Parisi, Salvatore Leotta, Alessandra Romano, Vittorio Del Fabro, Enrica Antonia Martino, Valeria Calafiore, Rachele Giubbolini, Uros Markovic, Valerio Leotta, Mary Ann Di Giorgio, Daniele Tibullo, Francesco Di Raimondo, Concetta Conticello

**Affiliations:** 1 Division of Hematology, AOU "Policlinico - Vittorio Emanuele”, Via Santa Sofia 78, 95124 Catania, Italy; marinaparisi@hotmail.it (M.S.P.); leotta3@yahoo.it (S.L.); vdelfabro@yahoo.it (V.D.F.); 2 Department of General Surgery and Medical-Surgical Specialties, Haematology Section, University of Catania, Via Santa Sofia 78, 95124 Catania, Italy; sandrina.romano@gmail.com (A.R.); enricaantoniamartino@libero.it (E.A.M.); valeriacalaf@gmail.com (V.C.); r.giubbolini10@gmail.com (R.G.); urosmarkovic09041989@gmail.com (U.M.); valerio_leotta@yahoo.it (V.L.); maryanndg@live.it (M.A.D.G.); diraimon@gmail.com (F.D.R.); 3 Department of Biomedical and Biotechnological Science, University of Catania, 95124 Catania, Italy

**Keywords:** multiple myeloma, double refractory, pomalidomide

## Abstract

Background: We retrospectively analysed relapsed/refractory MM (RRMM) patients treated with pomalidomide and dexamethasone (PomaD) either in real life, or previously enrolled in an interventional (STRATUS, MM-010) or currently enrolled in an observational study (MM-015) to provide further insights on safety and tolerability and clinical efficacy. Methods: Between July 2013 and July 2018, 76 RRMM patients (including 33 double refractory MM) received pomalidomide 4 mg daily given orally on days 1–21 of each 28-day cycle, and dexamethasone 40 mg weekly (≤75 years) or 20 mg weekly for patients aged > 75 years. In nine patients a third agent was added to increase the response: Cyclophosphamide (in two fit patients) or clarithromycin (in seven frail patients). Patients received subcutaneous filgrastim as part of the prophylaxis regimen for neutropenia. Results: A median number of six (range 2–21) PomaD cycles were given. The regimen was well tolerated with grade 3–4 haematological and non-haematological adverse events in 39 (51%) and 25 (33%) patients, respectively. In patients who developed serious AE, pomalidomide dose reduction (11%, 14%) or definitive discontinuation (18%, 23%) were applied. All patients have been evaluated for response within the first two cycles. The disease control rate (DCR), i.e., those patients that had a response equal or better than stable disease (≥ SD), was high (89%), with 44% overall response rate (ORR) after six cycles. The achieved best responses were complete remission (CR, 5%), very good partial remission (VGPR, 4%), partial remission (PR, 35%), minimal response (MR, 7%), and stable disease (SD, 38%). After a median follow up of 19.6 months, median progression free survival was 9.4 months, and overall survival (OS) was 19.02 months. Univariate analysis showed that double refractory patients, or who received more than three previous lines had shorter PFS. At 18 months, regardless of the depth of response, patients with a disease control of at least six months, defined as maintenance of a best clinical and/or biochemical response to treatment for almost six months, had prolonged PFS (35.3% versus 20.6%, *p* = 0.0003) and OS (81.2% versus 15.9%, *p* < 0.0001) Conclusions: Our findings indicate that PomaD is a safe and well-tolerated regimen in real-life, associated with prolonged PFS and OS with acceptable toxicity. Moreover, Pd induced disease control in most intensively pre-treated patients and some of them achieved longer PFS than that obtained with the previous treatment.

## 1. Introduction

In the last twenty years the outcome for multiple myeloma (MM) patients improved thanks to the introduction in clinical practice of new drugs such as immunomodulators (IMiDs), similar to thalidomide, lenalidomide, and proteasome inhibitors (PIs), including bortezomib and carfilzomib [1]. Second-generation novel agents, used alone or in combination, are effective and relatively safe. In this scenario, lenalidomide represented the backbone to combine with novel drugs, such as carfilzomib in the ASPIRE trial [2], daratumumab in the POLLUX trial [3], elotuzumab in ELOQUENT study [4], or ixazomib in the TOURMALINE trial [5]. However, most patients relapse and become refractory. Relapsed/refractory (RRMM) is defined as a disease which becomes non-responsive or progressive on therapy or within 60 days of the last treatment in patients who had achieved a minimal response (MR) or better on prior therapy [6]. Unfortunately, there are a few therapeutic options for patients who become refractory to both lenalidomide and bortezomib, defined ’double refractory’ (DRMM), with poor median survival [7].

Pomalidomide, the third-generation IMiD, is active against MM plasma cells, able to inhibit intra and extracellular mediators of proliferation, and to control bone marrow microenvironment by increasing immune response against MM plasma cells, inhibiting regulatory T-cells, and stimulating natural killer cells [8].

Pomalidomide has shown clinical activity in the multi-center randomized trial MM-003, which compared the combination of pomalidomide with either a low or high dose of dexamethasone [9] in RRMM who received at least two prior therapies including bortezomib and lenalidomide. In the MM-003 trial the median progression free survival (PFS) in the experimental arm with pomalidomide and dexamethasone was significantly higher than the control arm (four versus 1.9 months) with an overall response rate (ORR) of 32% versus 11% [10], as subsequently confirmed in the STRATUS-MM-010 trial [11]. 

Based on MM-003 trial results, EMA approved pomalidomide for RRMM patients that have received at least two prior therapies including bortezomib and lenalidomide, making pomalidomide and dexamethasone the backbone for standard third line therapy in Europe, and later in Italy in September 2015 [12]. Although all prospective randomized trials (MM-02; MM-03; MM-010; MM-013) have clearly demonstrated the role of PomaD in RRMM patients, real world experiences have a pivotal and additional role in describing the activity and safety of pomalidomide in patients that otherwise might not be represented in clinical trials. Indeed, the series enrolled in clinical trials usually include only few cases of DRMM, with patients having a sufficient performance status, an adequate hepatic and cardiac function, and an adequate bone marrow reserve. Therefore, the therapeutic outcome and the toxicity profile derived from a clinical trial may not entirely reflect the difficulties that can be encountered in treating a RRMM patient in a real-life setting [13]. 

A few single- and multi-institutions experiences in the real-life setting analyses have been recently published with variable rates of overall responses (ORR) and progression free survival [14] (Table 1).

We therefore retrospectively analysed the results in our single-centre series of 76 RRMM patients who received pomalidomide in combination with dexamethasone between 2013 and 2018 in order to provide further insights on the use of this salvage therapy.

## 2. Experimental Section

### 2.1. Patients and Treatments

From July 2013 to July 2018, 76 consecutive RRMM patients received pomalidomide and dexamethasone at the Division of Haematology, A.O. “Policlinico-Vittorio Emanuele”, University of Catania, Italy, as part of a national-named program (*N* = 47), enrolled in the single-arm phase IIIb MM-010 trial (*N* = 15) or in the observational phase IV MM-015 trial (*N* = 14). All patients except three had a measurable disease as defined by the International Myeloma Working Group (IMWG) guidelines and received at least two cycles of pomalidomide and dexamethasone.

The study was approved by the local institutional review board. All participants gave a written informed consent in accord to the Declaration of Helsinki. Basic characteristics and treatment are summarized in Table 2. Pomalidomide was given at 4 mg daily per os on days 1–21 of each 28-day cycle and dexamethasone 40 mg weekly (for <75 years patients) or 20 mg weekly (for ≥75 years patients) until progression. In nine patients (11.8%) (seven with a minimal response and two patients with only a stable disease), after two cycles a third agent was added in order to increase the response: Cyclophosphamide 50 mg per day for 10 days/cycle, in two fit patients, 2.6% and clarithromycin 500 mg bis in die for 21 days/cycle, in seven frail patients, 9%. Pomalidomide cycles were given until disease progression or unacceptable toxicity.

Concomitant medications included agents for thromboprophylaxis with low-dose aspirin for low risk patients and low-molecular-weight heparin for high risk patients and anti-infectious prophylaxis, consisting of Trimetoprim and Sulfametoxazole 800 mg bis in die only two days per week and Acyclovir 400 mg daily. Patients received subcutaneous filgrastim as part of the prophylaxis regimen when leukocytes count was ≤ 2.5 × 10^9^/L and neutrophils count was ≤ 1.5 × 10^9^/L. Supportive care with erythropoietin was given accordingly to ASH/ASCO guidelines [23]. Each patient’s medical history was recorded on day 1 of each cycle. Physical examinations were conducted, and blood was collected for haematology, renal, and liver function tests on day 1 of each course. FISH analysis was available in 26 patients before the PomaD start. Cytogenetic high risk was defined as the presence of, t (4; 14), t (14; 16), or del17p documented by FISH at any percentage level, according to the IMWG criteria [23]. 

### 2.2. Safety and Clinical Evaluation

Adverse events (AEs) were evaluated according to the National Cancer Institute Common Terminology Criteria for Adverse Events, version 4.0 [24]. Responses were evaluated according to the International Myeloma Working Group (IMWG) response criteria [25]. The clinical benefit rate (CBR) was considered the percentage of patients that had a response higher or equal to minimal response (≥MR); the disease control rate (DCR) included those patients that had a response equal or better than stable disease (≥SD).

### 2.3. Statistical Analysis 

Data were elaborated using GraphPad Prism version 6.00 for Windows, GraphPad Software, (manufacture, San Diego, CA, USA). Descriptive statistics were generated for analysis of results and *p*-value under 0.05 was considered significant. Patients-related (age, sex) and treatment-related (number of previous therapies, previous autologous, or allogenic transplantation) variables were compared with the variable "disease control for at least 6 months". Control of disease for at least six months is the maintenance of a best clinical and/or biochemical (CR, PR, VGPR, MR, or SD) response to treatment for almost six months. The comparison between these variables and the parameter control of disease for at least six months was evaluated in the univariate analysis. Fisher exact-test was used for nominal variables with two-categories; χ^2^ test for nominal variables with more than two categories. The variables that resulted significant from univariate analysis were evaluated in multivariate analysis in comparison with the variable control of disease for at least six months.

Overall survival (OS) was calculated from the time of inclusion until the date of death for any cause. Progression free survival (PFS) was defined as the time from the end of previous treatment to documented progression (defined as increase in monoclonal component in serum or urine of at least 25% from the baseline). Progression free survival obtained with the last previous treatment before pomalidomide was called PFS Pre-Poma. Progression free survival obtained with pomalidomide was called PFS Poma. 

OS and PFS were analysed by the Kaplan-Meier test. Standard errors were calculated by the method of Greenwood, the 95% confidence intervals are computed as 1.96 times the standard error in each direction. Survival analysis were performed by the Kaplan-Meier method. For the multivariate analysis, the logistic regression method was used. The statistical significance level was set at the 95th percentile. Significance above the 99th percentile was highly significant.

## 3. Results

### 3.1. Patients Characteristics

As shown in Table 2, half of the patients were male (*N* = 43, 56.5%), with a median age of 63 years (range 43–83 years); 66 (86.8%) patients had a secreting MM, seven (9.2%) had micromolecular and three (3.9%) a non-secreting MM. 

In most patients, the Durie and Salmon stage was higher or equal to IIA both at diagnosis (*N* = 70, 92%) and before PomaD treatment (*N* = 69, 90%); similarly, the ISS stage was higher or equal to II in 52 (68%) patients at diagnosis and in 58 patients (76%) at the time of PomaD treatment.

Renal function was assessed using the estimated glomerular filtration rate (eGFR) that was normal in 59 (77.6%) patients and severely compromised in four (5.2%), while 13 patients (17.1%) had an eGFR between 30 and 50 mL/min.

Bone lesions were present in all patients as detected by conventional skeletal survey, magnetic nuclear resonance (MNR), or PET analysis; 55 (72.3%) patients carried more than three lesions before the PomaD start.

A minority of patients had documented extra medullary disease (*N* = 10, 13.1%). Locations of extra medullary disease were different and insidious, including the peri-renal and peri-pancreatic (patient #3), sternal and acromion-clavear (patient #9), intracranial into clivus (patient #25), pulmonary (patient #26), sub-clavicular region from pleura (patient # 27), torsion and ileo-psoas muscle (patients #34 and #45), right thigh root (patient #37), chest wall (patient #44), left costal hypochondrium (patient #47) sites, as confirmed by imaging-guided biopsies in all the patients.

Results of FISH analysis showed the following alterations detected alone or in combination: Gain1q (seven patients, 9%), del 13q (12 patients, 16%), t (4; 14) (eight patients, 10.5%), t (11; 14) (eight patients, 10.5%), del 17p (eight patients, 10.5%). The ECOG performance status at relapse was higher or equal to two in 39 patients (51%).

Characteristics of MM disease and treatment exposure (number of previous lines of therapy, previous autologous, or allogenic transplantation) are summarized in Supplementary Table S1. The median time from first MM diagnosis was 67.5 months (range 19–239 months), with a median number of previous treatments was three (range 1–8): 36 (47.4%) patients were refractory to the last therapy and 40 (52.6%) were relapsed; last therapy before PomaD was an IMIDs-based regimen in 49 (64.4%) patients, a PIs-based regimen in 21 (27.6%) patients, and six (8%) patients had received conventional chemotherapy. Thirty-three (43%) patients were DRMM; 28 (36.8%) patients had received autologous transplantation, and eight (10.5%) of them had received allogeneic transplantation after autologous transplantation. 

### 3.2. Safety and Tolerability

Grade 3–4 hematologic or non-hematologic adverse events occurred respectively in 39 (51%) and 25 (32%) patients.

As shown in Table 3, the most frequent grade 3–4 haematological adverse events were: Neutropenia (*N* = 15, 19%), anaemia (*N* = 17, 22%), and thrombocytopenia (*N* = 6, 7.8%). The most frequent grade 3–4 non-haematological adverse events included: Infection (*N* = 7, 9%), glucose metabolism alteration (*N* = 5, 6.5%), sepsis (*N* = 4, 5%), pneumonia (*N* = 4, 5%), fatigue (*N* = 2, 2.6%), diffuse erythema (*N* = 2, 2.6%), thromboembolism (*N* = 2, 2.6%), diarrhoea (*N* = 1, 1.3%), hyponatremia (*N* = 2, 2.6%), neuropathy (*N* = 2, 2.6%), melena (*N* = 1, 1.3%), atrial flutter (*N* = 1, 1.3%), acute renal failure (*N* = 1, 1.3%). 

In patients with serious adverse events the dose of pomalidomide was reduced in 11 patients (14%) or temporarily interrupted in 12 (16%) and discontinued in 18 patients (23%). Safety and tolerability are shown in Table 3. 

### 3.3. Efficacy 

All patients were evaluable for response, having received at least two cycles of PomaD. The median of administered cycles was six (range 2–21). The disease control rate (DCR =≥ SD) was high (89%), with 44% overall response rate (ORR, =≥ PR, Table 4). The achieved best response was: Complete remission (CR, 5%), very good partial remission (VGPR, 4%), partial remission (PR, 35%), minimal response (MR, 7%), and stable disease (SD, 38%). No differences in the response rate were due to age, sex, baseline LDH, number of previous lines, reduced kidney function, or presence of extra-medullary disease. 

**Figure 1:**
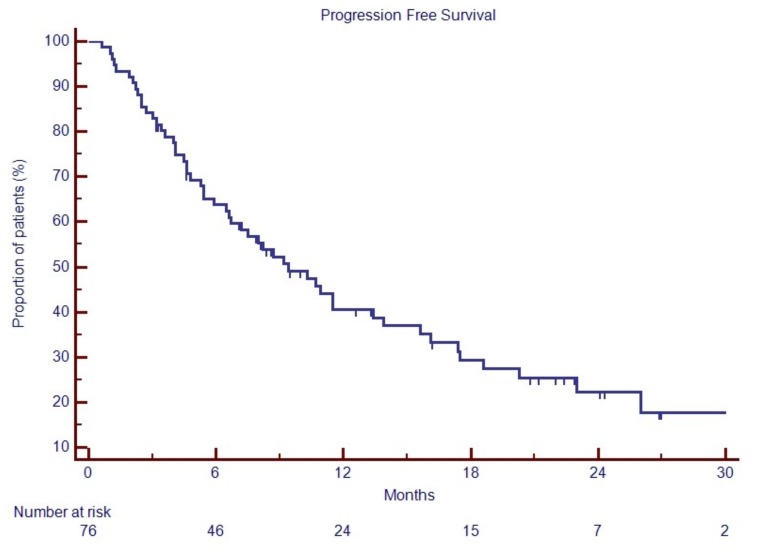

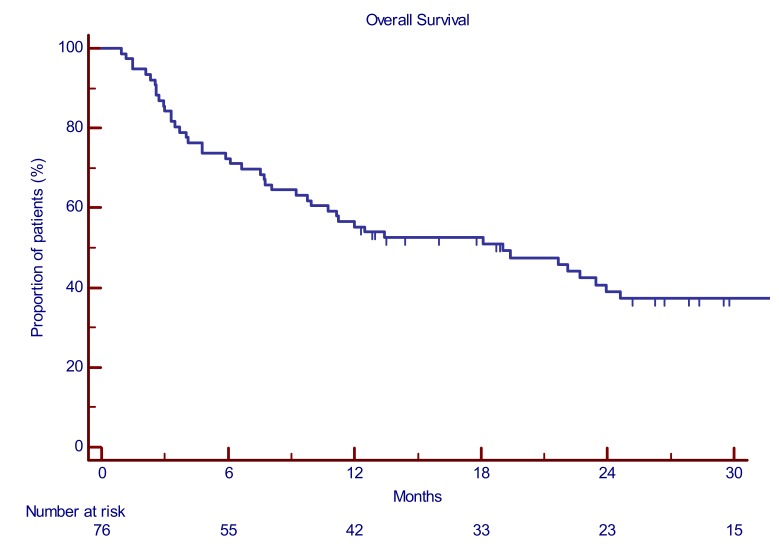
Efficacy of PomaD in RRMM.

After a median follow up of 19.6 months, median PFS was 9.4 months and median OS was 19.02 months, as shown in Figure 1. 

### 3.4. Progression Free Survival

Univariate analysis showed that double refractory patients, or who received more than three previous lines had shorter PFS, while extramedullary disease, clearance creatinine < 50 mL/min or the type of last treatment given (a regimen based on novel agents lenalidomide or bortezomib or conventional chemotherapy) did not have any impact on PFS (Table 5). Irrespective of the depth of response, the percentage of patients achieving disease-control of at least six months who were still progression-free surviving at 18 months was significantly higher with respect to patients who did not reach disease control at six months (respectively, 35.3% versus 20.6%, *p* = 0.003).

**Figure 2:**
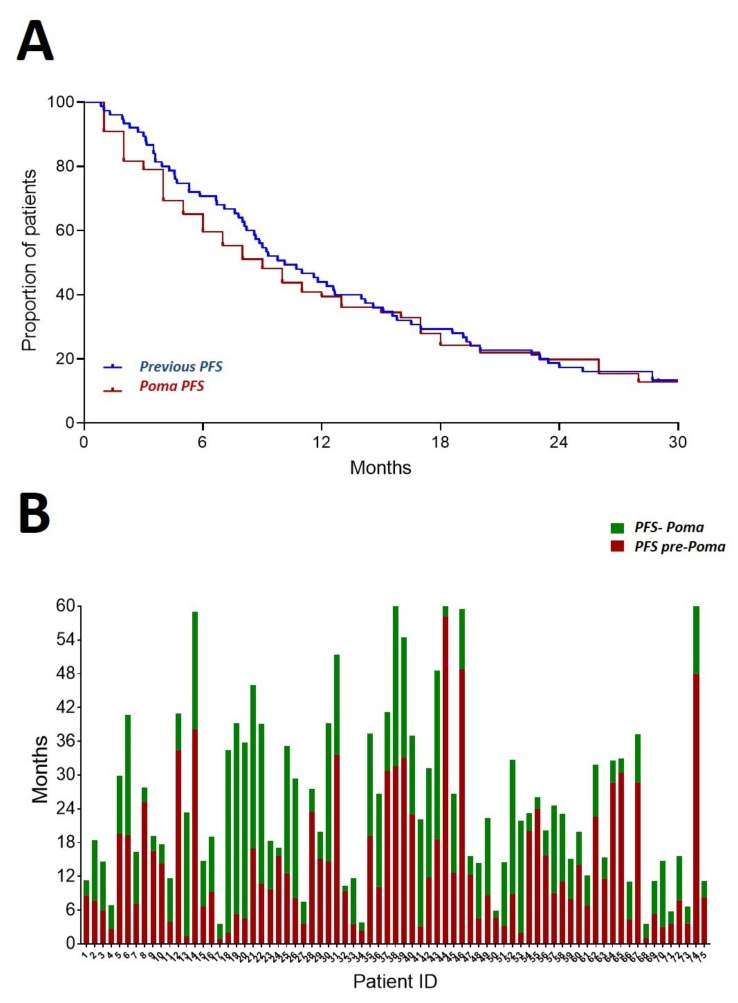
Progression free survival (PFS) before treatment with PomaD and with PomaD. (**A**) Kaplan-Meier curves for direct comparison of PFS before (blue) and after (red) PomaD start are shown; (**B**) PFS before (green) and after (red) PomaD start is shown for each individual patient of the study (data available for 75/76 patients).

Furthermore, we found that the median PFS before PomaD and after PomaD treatment were comparable (10.2 and 9.4 months, respectively) (Figure 2A) and in several patients the PFS after PomaD was longer than the PFS given by the previous regimen (Figure 2B). 

Multivariate analysis of PFS showed that double-refractoriness and control disease less than six months were independent predictors of inferior outcome (Table 6).

Univariate analysis of PFS and OS at 18 months in RRMM patients treated with PomaD (Table 5).

Multivariate analysis of PFS and OS at 18 months in RRMM patients treated with PomaD (Table 6).

### 3.5. PomaD Can Prolong Overall Survival Independently from the Quality of the Achieved Best Response

After a median follow-up of 19.6 months, 40/76 (52.6%) patients were alive. The median overall survival was 19.02 months. Based on the median number of administered cycles, we decided to evaluate the disease control after six cycles. Among all patients, 40 were still on treatment after six cycles, 30 progressed before, one patient discontinued treatment after three cycles because of lung carcinoma diagnosis, two patients interrupted treatment for adverse events, three patients were ongoing on the second or third cycle. Among those patients that were treated with PomaD in combination with a third drug, eight patients had a control of disease for almost six months, one progressed after two cycles.

Univariate analysis showed that patients receiving more than three previous lines of treatment had shorter OS, while extramedullary disease, use of pre-emptive G-CSF, increased LDH at the baseline, clearance creatinine < 50 mL/min, or the type of last treatment given (a regimen based on novel agents lenalidomide or bortezomib or conventional chemotherapy) did not have any impact on OS (Table 5). Patients who achieved PR within the first six cycles had longer OS than those who do not achieve PR (33.7% versus 24.6%, *p* = 0.03). Patients with a disease control of at least six months, defined as maintenance of a clinical and/or biochemical response to treatment for almost six months, had prolonged OS (56.3% versus 23.3%, *p* < 0.0001). Similarly, patients with a disease control of at least 12 months had longer PFS and OS than those with a disease control < 12 months (Table 5).

## 4. Discussion

Herein, we described our single-centre experience in treating RRMM patients with PomaD. The drug was introduced for treatment of RRMM patients when the therapeutic options for RRMM were limited, thus representing a valid choice, especially for unfit patients who could not receive parenteral treatment at the hospital [26]. Nowadays, the largest real-world multicentric series includes 94 evaluable patients (33% refractory) treated with PomaD in 12 Italian haematological centers between 2016 and 2018. Median TTNT was 30 months and ORR was 51%. Age > 70 years, haematological response, and previous therapies did not influence OS although the frailty score and high serum LDH did [27]. Another large study includes 87 evaluable patients (69% double refractory) [22], treated with PomaD in Australia between 2010 and 2015. After a median follow-up of six months, the overall response rate was 32% with a median PFS of 3.4 months and a median OS of 7.5 months [22]. Age < 65 years, male sex, hemoglobin <100 g/L, and platelets < 100 × 10^9^/L values had statistical significance for PFS whereas age < 65 years, double refractory status, hemoglobin < 100 g/L, and platelets < 100 × 10^9^/L were significant for OS.

In a British series of 39 patients, including one third of DRMM, the ORR was 41%, with a median PFS of 5.2 months, and OS of 13.1 months [17]. Despite the dismal outcome, Sriskandarajah et al. suggested that in some patients PomaD could maintain durable response rather than depth response, translating in longer overall survival [17]. A recently published unicentric French study on 63 patients reported, after a median follow up of 28 months, an ORR of 51% and a median OS of just 6.4 months in patients with early discontinuation of treatment versus 26.8 months in patients with SD, and not reached in responders’ patients [28]. A better response rate was described by Charlinski et al. but in this series 18% of patients were treated with pomalidomide, dexamethasone, and bortezomib [29].

The baseline characteristics of the patients included in our study were comparable to those reported in the MM-003 trial [12,28] and other real-world series. In our experience, the ORR was 44%, and after a median follow-up of 19.6 months, we found both median PFS and OS longer than those reported in literature (Table 1). This could be due to the real-world practice of continuing treatment despite a biochemical progression if there is a subjective clinical benefit. Indeed, a post hoc analysis of MM-003 trial that investigates a possible correlation between OS and response status (SD, PD or >PR) has demonstrated that PomaD provided clinical benefit in SD patients, with similar survival results compared to responding patients and much better survival than progressing patients [12,28]. Based on the frailty, in some patients a third drug was added based on previous reports [30]: Cyclophosphamide for fit patients, and clarithromycin in frail patients. Among these patients that were treated with PomaD in combination with cyclophosphamide or clarithromycin, eight patients had a control of disease for almost six months, one (belonging to the cyclophosphamide group) progressed after two cycles. None of these patients had an extramedullary disease, and, for those seven patients treated with cyclophosphamide, median PFS was eight months.

Pomalidomide and dexamethasone combination was feasible and well tolerated. In the main clinical trials in which the use of pomalidomide in RRMM patients has been studied (MM02, MM03, and MM10), the most frequent haematological adverse event of grade 3/4 was neutropenia (between 41% and 50%) followed by anaemia (22% to 33%) and thrombocytopenia (between 19% and 24%) [12]. In our hands, grade 3/4 neutropenia was recorded only in 19% of patients, probably due to a GSF-based primary prophylaxis [31].

The most frequent non-haematological grade 3/4 adverse events were pulmonary infections (between 10% and 22%) and fatigue (between 6% and 14%), while thromboembolic events were rare.

In patients who developed serious adverse events, the dose of pomalidomide was reduced (14%) or discontinued (23%), in percentages different from those registered in trial settings [12,32]. In the MM-010 trial, pomalidomide was reduced in one third of patients and treatment was discontinued for adverse events in less than 5%. Similarly, in the MM03 trial, 30% of patients reduced pomalidomide, but in 67% of cases, treatment was temporarily interrupted and discontinued in about 6% of the cases. In the MM02 study, dose reduction occurred in 22% of patients and temporary interrupted in 67% of patients; 6% of patients permanently discontinued treatment for adverse events. Given the presence of additional salvage regimens in our setting (including carfilzomib [33] and daratumumab based regimens) the percentage of patients who discontinued treatment is higher than historical series. Treatment of frail RRMM patients is challenging. In our series, renal impairment and age did not affect efficacy or toxicity, confirming data from three pooled trials. Our analysis suggest that the most relevant predictor of outcome is the six-months disease control, independently form the depth of response. Interestingly, almost half of patients obtained a PFS which exceeded the PFS obtained with previous treatment, reverting the typical clinical course of MM characterized by decreased durability of response at each successive salvage regimen [19]. In all real-life experiences [16,18,19,34,35,36,37], there is a sub-group of patients in which continued exposure to poma resulted in stable disease that can be translated in survival prolongation. Most of these patients achieved only a minimal-partial response demonstrating the relevance of maintaining response rather than deepening response in a specific cohort of patients. 

Therefore, a new information is emerging from these studies where the combination of pomalidomide and dexamethasone is able to keep the disease under control albeit not inducing a deep remission. In this perspective, our study is a further confirmation of this pomalidomide activity since we have found that patients who achieve a control of disease for more than six months have a prolonged PFS and OS irrespective of the deepness of response. In our study, median OS was much higher than in registrative studies and other real-life studies. We think that the reasons for this achievement rely only in part on the fact that our patients were less heavily pre-treated (median number of previous treatments was three in our series versus five lines in registrative studies). The main reason we believe is the strong supportive therapy that accompanied the PomaD treatment in our patients. Prophylaxis with trimethoprim, sulfametoxazole, and acyclovir has certainly reduced the incidence of infectious episodes and especially pneumonia that represents the main cause of death and therapy discontinuation in most series. More important, in our patients, we adopted an aggressive policy of growth factor administration that allowed us to keep on treatment most patients. In many series, severe neutropenia was the most frequent toxicity ranging from 31% to 55% and was responsible for interruption of treatment or reduction of the dose of pomalidomide. Our patients experienced severe neutropenia only in 19% of cases and it has been possible to maintain patients on treatment in most cases. Therefore, even though most patients had a minimal response, they were able to continue the treatment and consequently to obtain a long control of the disease. In this perspective, it should be underlined that a study that evaluated different dosages and schedules with a long follow up, suggests that a longer duration of treatment together with the best response rates and fewer AEs are obtained with the 2 mg pomalidomide dose [18]. 

## 5. Conclusions

Several novel categories of drugs and old combinations with different drugs are available and show efficacy in heavily pre-treated RRMM patients. However, these patients complain an increasing frailty because of the cumulative toxicities from the previous treatments. In this scenario, a therapeutic option that warranties a sufficient control of the disease together with a good tolerability seems to have a central role in the management of this category of patients. Our study confirms that PomaD in RRMM is able to control the disease with limited and manageable side effects, even though in the absence of deep responses thus supporting its use especially in the third line of treatment. 

## Figures and Tables

**Table 1:** Efficacy of pomalidomide and dexamethasone (PomaD) in real-world practise.

Study Design	Nation	Number of Patients Involved	Percentage of Double Refractory Patients	ORR (%)	Median PFS (Months)	Median OS (Months)	References
Multi-centric (5)	UK	85 (70)	72.9	52.9	5.2 (13.2)	13.7 (13.2)	Macrocia, BJH 2017 [15]
Uni-centric	UK	39	33	41	5.2	13.1	Sriskandarajah, leuk&lymph 2016 [16]
Uni-centric	France	63	19	51		6.4-26.8-nr	Gueneau, Eur J Hematol 2018 [17]
Multi-centric	Poland	50	26	31.6–75 (plus Bortezomib)	9,5	14	Charlinski, Eur J Hematol 2018 [18]
Multi-centric	Australia	151 (87)	69	32	3.4	7.5	Scott leuk&lymphoma 2018 [19]
Uni-centric	India	24	75	50	6		Jandial Leukemia & lymphoma 2018 [20]
Kansai Myeloma Forum (multi-centric)	Japan	108	54	31.3	4.4 (median Time to Treatment Failure)	Nr	Matsumura-Kimoto Int JH 2018 [21]
Multi-centric	Italy	103 (94)	33 % refractory	51%	30 (mTTNT)	16	Mele Leukemia and Lymphoma 2019 [22]
Uni-centric	Italy	76	43	44	9.4	19.02	present study

**Table 2:** Patients’ clinical characteristics (Cytogenetic high risk was defined as the presence of, t (4; 14), t (14; 16), or del17p documented by FISH).

**Age**	
Median (range)	63 (43-83)
<61 years, *N* (%)	29 (38.1%)
61–71 years, *N* (%)	32 (42.1%)
>71 years, *N* (%)	15 (19.7%)
**Gender**	
Male, *N* (%)	43 (56.5%)
Female, *N* (%)	33 (43.4%)
**Paraprotein (isotype)**	
secreting, *N* (%)	66 (86.8%)
micromolecolar, *N* (%)	7 (9.2%)
non secreting, *N* (%)	3 (3.9%)
Kappa–light chain, *N* (%)	44 (60.2%)
Lambda–light chain, *N* (%)	29 (39.7%)
**ECOG (Performance Status at baseline)**	
0–1, *N* (%)	37 (48.6%)
2, *N* (%)	29 (38.1%)
3 or more, *N* (%)	10 (13.1%)
**Durie and Salmon Stage at Baseline**	
IA, *N* (%)	7 (9.2%)
IIA, *N* (%)	19 (25%)
IIIA, *N* (%)	41 (53.9%)
IIB, *N* (%)	1 (1.3%)
IIIB, *N* (%)	8 (10.5%)
**ISS Stage at Baseline**	
I, *N* (%)	18 (23.6%)
II, *N* (%)	21 (27.6%)
III, *N* (%)	37 (48.6%)
**Risk Class at Relapse According to IMWG (26pts)**	
High, *N* (%)	9 (34.6%)
Standard, *N* (%)	17 (65.4%)
**Creatinine Clearance**	
<30 mL/min, *N* (%)	4 (5.2%)
30–50 mL/min, *N* (%)	13 (17.1%)
>50 mL/min, *N* (%)	59 (77.6%)
**Bone Lesions**	
At least 3, *N* (%)	55 (72.3%)
Less than 3, *N* (%)	21 (27.6%)
**Extramedullary Lesions**	
Yes, *N* (%)	10 (13.1%)
No, *N* (%)	66 (86.8%)

FISH: Fluorescence In Situ Hybridization. ECOG: Eastern Cooperative Oncology Group. IMWG: International Myeloma Working Group.

**Table 3:** Tolerability, treatment exposure, and adverse events in relapsed/refractory MM (RRMM) patients treated with PomaD.

Exposure/Tolerability	
mean duration, months (range)	7.2 (2–21)
dose reduction, *N* (%)	11 (14%)
dose interruption, *N* (%)	18 (23%)
deaths (no treatment-related), *N* (%)	28 (50%)
hematological adverse events (grade 3–4), *N* (%)	39 (51%)
neutropenia, *N* (%)	15 (19%)
anemia, *N* (%)	17 (22%)
thrombocytopenia, *N* (%)	7 (8%)
non-hematological adverse events (grade 3–4), *N* (%)	25 (32%)
infection, *N* (%)	7 (9%)
glucose metabolism alteration, *N* (%)	5 (6.5%)
sepsis, *N* (%)	4 (5%)
pneumonia, *N* (%)	4 (5%)
fatigue, *N* (%)	2 (2.6%)
diffuse erythema, *N* (%)	2 (2.6%)
thromboembolism, *N* (%)	2 (2.6%)
diarrhea, *N* (%)	1 (1.3%)
hyponatremia, *N* (%)	2 (2.6%)
neuropathy, *N* (%)	2 (2.6%)
melena, *N* (%)	1 (1.3%)
atrial flutter, *N* (%)	1 (1.3%)
acute renal failure, *N* (%)	1 (1.3%)

**Table 4:** Evaluation of efficacy of PomaD in RRMM.

	Within First 6 Cycles, *N* (%)	Best Response, *N* (%)	
**CR**	3 (4)	4 (5)	ORR 44% DCR 89%
**VGPR**	3 (4)	3 (4)	
**PR**	19 (25)	27 (35)	
**MR**	10(13)	5 (7)	
**SD**	33 (43)	28 (38)	
**PD**	8 (11)	8 (11)	

Response rate after six cycles and best response rates are reported. Abbreviations: CR: Complete remission; VGPR: Very good partial remission; PR: Partial remission; SD: Stable disease; PD: Progression disease; MR: Minimal response; ORR: Overall response rate; DCR: Disease control rate.

**Table 5:** Univariate analysis of PFS and overall survival (OS) at 18 months in RRMM patients treated with PomaD.

		*N*	PFS@ 18 Months (% Survival)	*p*-Value	OS@ 18 Months (% Survival)	*p*-Value
age	≤65	49	27.8	0.98	57.1	0.64
	>65	27	36.1		56.8	
sex	Male	43	28.9	0.86	57.2	0.33
	Female	33	30.3		55.4	
extramedullary disease	No	66	29.4	0.86	54.3	0.77
	Yes	10	30.0		70.1	
baseline LDH	normal	47	18.9	0.21	70.1	0.43
	increased	29	32.5		58.1	
G-CSF	No	36	23.1	0.21	63.2	0.95
	Yes	40	36.1		49.1	
prior lines	3	22	49.6	0.0001	57.3	0.53
	more than 3	54	17.6		53.2	
last therapy before Pd start	lenalidomide	49	37.2	0.77	60.2	0.12
	bortezomib	20	25.0		65.1	
double refractory	no	43	43.3	*0.002*	67.7	0.08
	yes	33	10.8		44.0	
disease control	<6 months	40	20.6	*0.003*	15.9	*<0.0001*
	>6 months	36	35.3		81.2	
disease control	<12 months	47	0.0	*0.0003*	10.3	*<0.0001*
	>12 months	29	29.5		88.3	
ClCr	<50 mL/min	13	21.6	0.12	56.2	0.81
	>50mL/min	63	28.5		55.8	
Response at 6 cycles	less than PR	41	25.1	*0.03*	43.1	*0.01*
	At least PR	35	63.9		72.3	

PFS: Progression Free Survival. OS: Overall Survival. LDH: lactate dehydrogenase. Pd: Pomalidomide and desamethasone.

**Table 6:** Multivariate analysis of PFS and OS at 18 months in RRMM patients treated with PomaD.

		PFS, HR, (95% CI)	*p*-Value	OS, HR, (95% CI)	*p*-Value
double refractory	no	2.85	0.0004	NA	
	yes	(1.60–5.11)			
disease control	<6 months	0.43	0.02	0.43 (0.25–0.84)	0.02
	>6 months	(0.25–0.84)			
disease control	<12 months	0.54 (0.21–1.45)	0.22	NA	
	>12 months				
prior lines	3	0.48	0.06	NA	
	more than 3	(0.22–1.03)			
response at 6 cycles	less than PR	0.53	0.22	NA	
	At least PR	(0.20–1.44)			

PFS, HR: Progression Free Survival, Hazard Ratio. OS, HR: Overall Survival, Hazard Ratio.

## Supplementary Materials

The following are available online at https://www.mdpi.com/2077-0383/8/10/1695/s1, Table S1: Previous treatments in a cohort of 76 RRMM patients treated with PomaD.
