# Supplementary material for: Clinical Benefit of Long-Term Disease Control with Pomalidomide and Dexamethasone in Relapsed/Refractory Multiple Myeloma Patients
Source: J Clin Med. 2019 Oct 16;8(10):1695. doi: 10.3390/jcm8101695 (PMC6832641; doi:10.3390/jcm8101695)
Supplement: Supplementary file 1 [file jcm-08-01695-s001.pdf]

Supplementary Table S1. Previous treatments in a cohort of 76 RRMM patients treated with PomaD.

**Median of previous treatments (range)**	3 (1-8)
**Median time between diagnosis and baseline**	65.9 months
**State of disease before pomalidomide treatment**	
Refractory, N (%)	36 (47.3%)
Relapse, N (%)	40 (52.6%)
Double refractory, N (%)	33 (43%)
**Last therapy before pomalidomide treatment**	
IMIDs, N (%)	49 (64.4%)
PIs, N (%)	21 (27.6%)
Conventional chemotherapy, N (%)	6 (8%)
**Previous autologous transplantation**	28 (36.8%)
**Previous allogenic transplantation**	8 (10.5%)
